# Severe housing cost burden and premature cardiovascular mortality

**DOI:** 10.1016/j.ajpc.2025.101021

**Published:** 2025-06-02

**Authors:** Nishad S. Kosaraju, Tanisha Choudhury, Lee Stoner, Aleah L. Thomas, Kaitlin E. White, Marcus R. Andrews, Briana I. Lawrence, Cameron K Ormiston, Lee Mason, Meredith S. Shiels, Aldenise P. Ewing, Yingxi Chen, Jennifer K. McGee-Avila, Wayne R. Lawrence

**Affiliations:** a Edward Via College of Osteopathic Medicine – Carolinas Campus, Spartanburg, SC, USA; b Division of Cancer Epidemiology and Genetics, National Cancer Institute, National Institutes of Health, Rockville, MD, USA; c College of Arts and Sciences, University of North Carolina Chapel Hill, Chapel Hill, NC, USA; d Department of Exercise and Sport Science, University of North Carolina at Chapel Hill, Chapel Hill, NC, USA; e Department of Epidemiology, The Gillings School of Global Public Health, University of North Carolina at Chapel Hill, Chapel Hill, NC, USA; f Center for Health Promotion and Disease Prevention, University of North Carolina at Chapel Hill, Chapel Hill, NC, USA; g Weill Cornell Medicine, New York, NY, USA; h Social Determinants of Obesity and Cardiovascular Risk Laboratory, National Heart, Lung, and Blood Institute, National Institutes of Health, Bethesda, MD, USA; i School of Medicine, Tufts University, Boston, MA, USA; j Department of Medical Education, Icahn School of Medicine at Mount Sinai, New York, NY, USA; k Division of Epidemiology, College of Public Health, The Ohio State University, Columbus, OH, USA

**Keywords:** Housing, Medicaid, Cardiovascular mortality, Premature death, Cardiovascular Disease

## Abstract

**Background:**

The proportion of people living in unaffordable housing in the U.S. has grown, and studies have documented a relationship between housing cost burden and poor cardiovascular health. We investigated the association between severe housing cost burden (SHCB) and premature mortality due to cardiovascular disease (CVD) and its subtypes overall and by sex. We further evaluated whether Medicaid expansion status moderated the association between SHCB and premature CVD mortality.

**Methods:**

We linked county-level SHCB data from the 2016–2020 American Community Survey with mortality data ascertained from national death certificate data. SHCB was measured as the percentage of households that spend ≥50 % of their income on housing and was categorized into distribution-based quintiles (1=lowest and 5=highest). States were classified based on Medicaid expansion status (expanded, late expanded, non-expanded). Multilevel-linear mixed models, adjusting for confounders, were used to estimate the adjusted rate ratios (aRR) for the association between SHCB and premature CVD mortality.

**Results:**

The highest SHCB quintile, compared to the lowest, had a 15 % higher premature CVD mortality rate (aRR=1.15; 95 %CI 1.06–1.24). Among men, the highest quintile of SHCB had a higher premature mortality rate due to ischemic heart disease (aRR=1.09; 95 %CI 1.01–1.17) and stroke (aRR=1.19; 95 %CI 1.06–1.32) compared with the lowest quintile. Compared to Medicaid expanded states, non-Medicaid expanded states had higher rates of premature CVD mortality for each SHCB quintile (Quintile 5: aRR=1.19; 95 %CI 1.02–1.36).

**Conclusion:**

Our findings suggest counties with greater SHCB, especially if situated within a non-Medicaid expansion state, have higher rates of premature CVD mortality.

**Figure:**
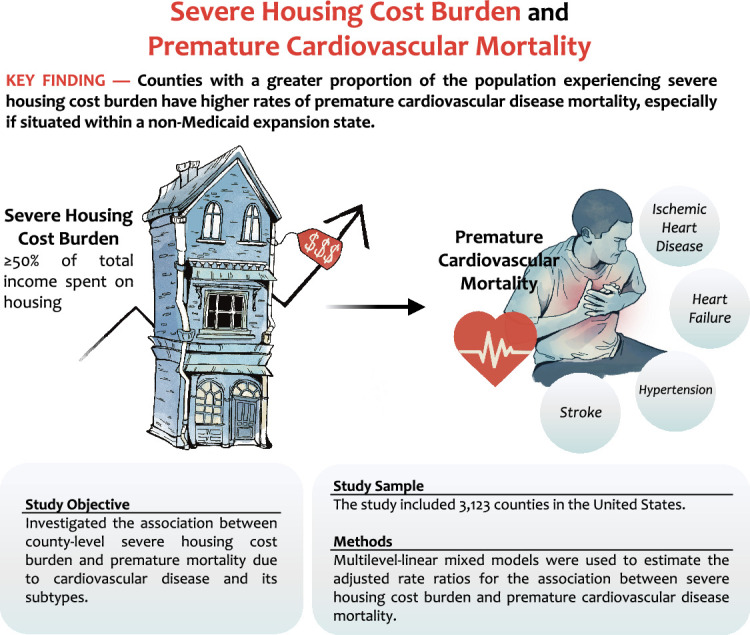
Central Illustration.

## 1 Introduction

Unaffordable housing in the United States (U.S.) has increased substantially, disproportionately affecting low-income families [1]. Severe housing cost burden (SHCB), defined as households that spend at least 50 % or more of their income on housing, has increased in recent years [2]. In 2021, >8 million households in the U.S. were experiencing SHCB [2]. The financial strain of unaffordable housing forces a detrimental trade-off, where individuals forgo necessary medical care to meet basic living expenses, resulting in poorer health outcomes [3,4]. An emerging body of research has suggested a relationship between housing unaffordability and poor cardiovascular health [[5], [6], [7]]. Therefore, among individuals with cardiovascular disease (CVD), housing unaffordability and related barrier to care may contribute to increased risk of premature death from CVD [5,6].

The Patient Protection and Affordable Care Act provided states with the ability to expand Medicaid coverage to additional low-income individuals, including childless adults, most of whom were previously excluded [8]. Expansion of Medicaid has been shown to reduce healthcare-related financial burdens, and associated with a reduction in the rate of home evictions [9,10]. Studies have reported that Medicaid expansion reduces out-of-hospital deaths related to CVD, and >10 % of Medicaid-insured adults <64 years of age have a history of heart disease [11]. Previous research has suggested that Medicaid expansion resulted in higher rates of provision of cardiovascular medications and greater utilization of cardiovascular-related preventive care [12,13]. However, the combined influence of Medicaid expansion and SHCB on premature mortality from CVD remains poorly understood.

In this ecological study, we investigated the association between SHCB and rates of premature mortality from CVD. We further evaluated whether the association was moderated by state Medicaid expansion status.

## 2 Methods

Demographic characteristics and CVD mortality rates overall and by leading subtypes (ischemic heart disease, heart failure, hypertension, stroke) were ascertained from the National Center for Health Statistics from January 2016 to December 2020. The International Statistical Classification of Diseases and Related Health Problems, 10th Revision codes for each outcome are presented in **Supplemental Table 1**. To focus on premature CVD mortality, our analysis was restricted to people aged 25–64 years, aligning with prior studies [14,15]. The National Institutes of Health Institutional Review Board waived approval and informed consent because the study used publicly available deidentified data. This study followed the Strengthening the Reporting of Observational Studies in Epidemiology (STROBE) reporting guidelines.

### 2.1 Severe housing cost burden

County-level SHCB data was obtained from the 2016–2020 American Community Survey. SHCB was defined as the percentage of households whose housing expenses accounted for ≥50 % of their total household income [16]. SHCB was categorized into distribution-based quintiles, where higher quintiles represented counties where a greater percentage of the population experienced SHCB.

### 2.2 Covariates

The county-level percentages of population below 150 % of the poverty line, uninsured adults, and non-Hispanic White alone race were obtained from the 2020 American Community Survey [16]. The county percentage of population below 150 % of the poverty line was based on the ratio of income to poverty-level in the past twelve months [16]. County-level education was measured using the education index, which is the percentage of people ages ≥25 years that have less than high school graduate (did not graduate), high school graduate only, and more than high school graduate [16,17]. Rurality of counties was based on the Rural-Urban Continuum Codes developed by the U.S. Department of Agriculture [18].

### 2.3 Medicaid expansion status

We categorized states according to their Medicaid expansion status as 1) Medicaid expanded (implemented the Patient Protection and Affordable Care Act Medicaid expansion on or before January 1, 2014 [*N* = 25]); 2) late expansion states (implemented Medicaid expansion between January 2, 2014 and December 31, 2019 [*N* = 12]); and 3) non-Medicaid expanded states (*N* = 14) (**Supplemental Table 2**).

### 2.4 Statistical analyses

Age-adjusted premature CVD death rates by SHCB overall and by Medicaid expansion status, and sex were calculated using direct standardization. All rates were age-standardized in five-year age groups to the 2000 U.S. population. To compare CVD death rates overall and by subtype and sex, we used age-standardized death rates from 2016–2020. Additionally, we calculated overall and by subtype and sex the adjusted mortality rate ratios (aRRs) and 95 % confidence intervals (95 %CI) of each quintile group compared with the first quintile group (reference), with multilevel linear mixed models weighted by county population, clustered at the state-level, and adjusted for metropolitan status, proportion of the population 150 % below poverty level, proportion of the population with non-Hispanic White alone race, proportion of uninsured adults, and education index. We further calculated aRRs comparing non-Medicaid expanded states to Medicaid expanded (referent) by quintile of SHCB. *P* for trends were calculated by modeling quintiles of SHCB as a continuous variable. All analyses were conducted using SAS version 9.4 (SAS Institute Inc, Cary, NC).

## 3 Results

**Fig. 1:**
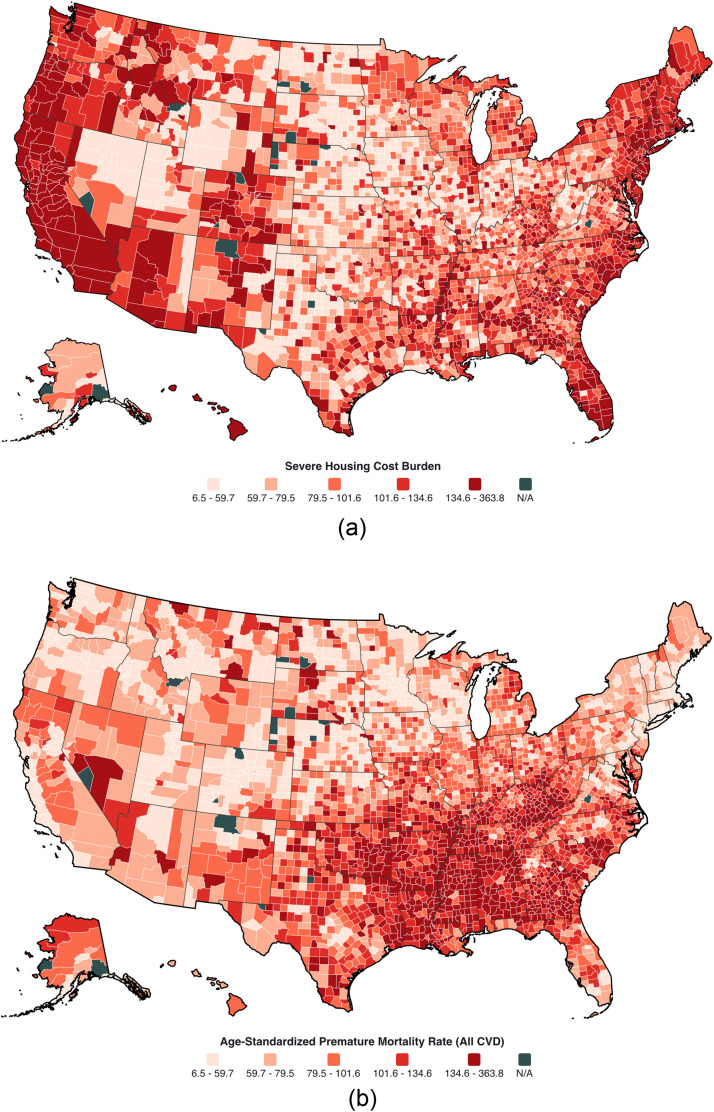
Severe housing cost burden and premature mortality due to major cardiovascular disease in the US, 2015–2020. (A) Counties by severe housing cost burden in quintile, and (B) by age-adjusted premature mortality rates due to cardiovascular disease by quintile.

The study included 3123 counties. Counties with greater SHCB were clustered in more metropolitan counties, and premature CVD mortality was greater in the southern region (Fig. 1). Overall, the age-adjusted premature CVD mortality rates per 100,000 population were higher for the lowest quintile of SHCB compared with the highest (Quintile 1 = 82.5 vs Quintile 5 = 74.6 per 100,000; P_trend_ <0.001) (**Supplemental Figures 1**). The age-adjusted premature CVD death rates were greater among men than women across quintiles of SHCB (Quintile 5 Men=104.9 vs Women=46.0 per 100,000). Similar results were observed when examined by CVD mortality subtype (**Supplemental Figure 2**).

**Fig. 2:**
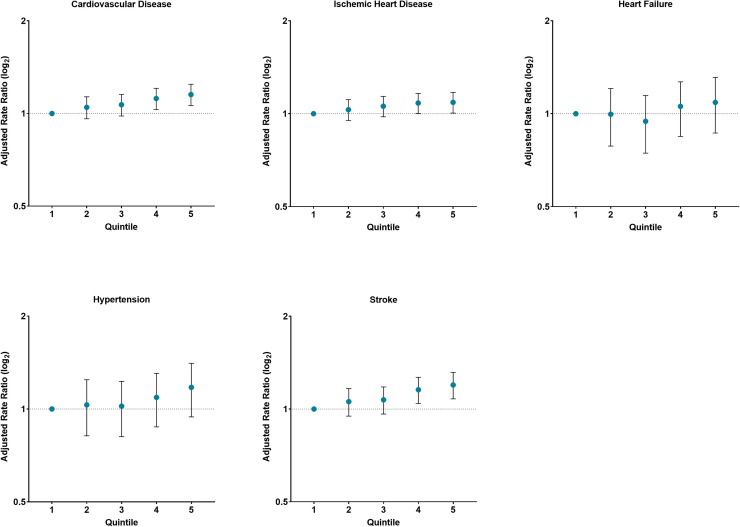
Adjusted premature cardiovascular mortality rate ratios by quintile of severe housing cost burden in U.S. counties, 2016–2020 Adjusted for metropolitan status, proportion 150 % below poverty level, proportion of the population with non-Hispanic White alone race, proportion of uninsured adults, and education index (percent with less than high school graduate, high school only and more than high school among ages ≥25 years). Note: The first quintile was used as the reference group As quintiles increase the larger the percentage of county households with severe housing cost burden.

After adjusting, counties in the highest quintile had a 15 % higher premature CVD mortality rate compared with counties in the lowest quintile (aRR=1.15; 95 %CI 1.06–1.24; P_trend_<0.001) (Fig. 2). Similar findings were observed by CVD mortality subtype for ischemic heart disease (aRR=1.09; 95 %CI 1.01–1.17; P_trend_<0.001), and stroke (aRR=1.20; 95 %CI 1.08–1.32; P_trend_<0.001). The premature CVD mortality aRRs was similar between men and women in counties in the highest quintile of SHCB compared with their counterparts in the lowest (Men: aRR=1.15; 95 %CI 1.07–1.24 and Women: aRR=1.15; 95 %CI 1.04–1.26). In analysis by CVD mortality subtype, men in the highest quintile of SHCB had a 9 % higher ischemic heart disease and 19 % higher stroke premature CVD mortality rates compared with the lowest quintile (ischemic heart disease: aRR=1.09; 95 %CI 1.01–1.17; P_trend_<0.001 and Stroke: aRR=1.19; 95 %CI 1.06–1.32; P_trend_<0.001) (**Supplemental Figure 3**). No associations were observed by CVD subtype among women (**Supplemental Figure 4**).

**Fig. 3:**
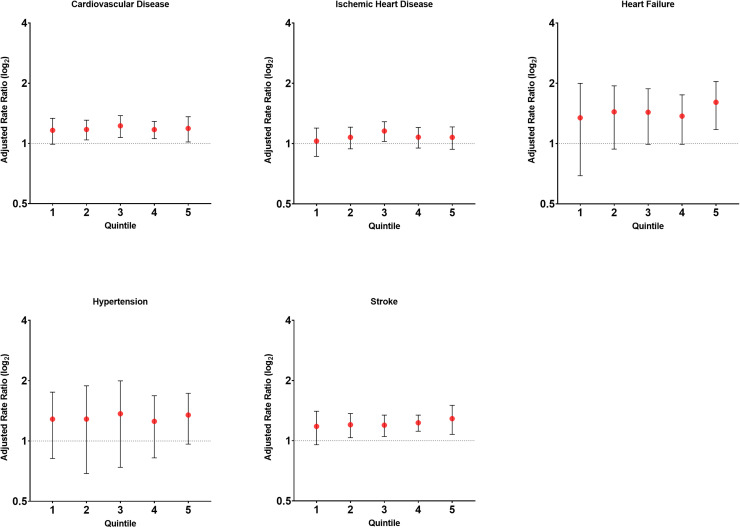
Adjusted premature cardiovascular mortality rate ratios by quintile of severe housing cost burden and state medicaid expansion status in U.S. counties, 2016–2020 Adjusted for metropolitan status, proportion 150 % below poverty level, proportion of the population with non-Hispanic White alone race, and education index (percent with less than high school graduate, high school only and more than high school among ages ≥25 years). Note: Medicaid expanded states were used as the reference group. Late Medicaid expanded states were excluded. As quintiles increase the larger the percentage of county households with severe housing cost burden.

Overall, compared with counties in Medicaid expanded states, non-Medicaid expanded states had higher rates of premature CVD mortality across higher quintiles of SHCB (Quintile 5: aRR=1.19; 95 %CI 1.02–1.36) (Fig. 3). Similar findings were observed for the highest quintile of SHCB for heart failure (aRR=1.61; 95 %CI 1.18–2.04) and stroke (aRR=1.29; 95 %CI 1.08–1.50).

## 4 Discussion

In this study, counties in the highest quintile of SHCB had higher premature CVD mortality, and the associated aRRs were largely greater among men and in non-Medicaid expanded states. Furthermore, most CVD subtypes showed significant incremental increases in association between greater county SHCB and higher premature mortality.

Interestingly, the age-adjusted premature CVD mortality rates were slightly greater in the lowest quintile of SHCB compared with the highest quintile. However, after adjusting for county-level characteristics, we observed a significantly higher rate among the highest quintile compared with the lowest quintile. These findings suggest that county-level characteristics have a large effect on the relationship between SHCB and premature CVD mortality. One potential mechanism for our findings is that individuals experiencing unaffordable housing are more likely to ration or postpone seeking preventative medical care because of housing-related financial obligations [1,3]. Also, prior research has linked housing instability to poor cardiovascular health, where the financial strain of SHCB may elevate risk of premature CVD mortality [5,6]. Additionally, the association between SHCB and premature CVD mortality was observed among both men and women, though patterns differed by sex. Overall, men and women had similar increased premature CVD mortality rates as quintiles of SHCB increased. However, for ischemic heart disease and stroke, men experienced greater increases in mortality rates as quintiles of SHCB increased. Prior studies have suggested social- and behavioral-related cardiovascular risk factors are contributors to greater risk of CVD at younger ages among men than women, potentially explaining current findings [19,20]. Previous research has reported that the relationship between economic strain and adverse health, including cardiovascular event, was more pronounced among men [21,22]. Further research is needed to investigate whether men are more vulnerable than women to the adverse impact of financial strain (i.e., SHCB) and related stressors on cardiovascular health.

We observed that Medicaid expansion may play a role in reducing premature CVD mortality among counties with a greater proportion of households experiencing SHCB. These findings may reflect the significant barriers to care uninsured or underinsured individuals in non-Medicaid expanded states encounter, including greater out-of-pocket medical costs and reduced preventive care access and utilization [[23], [24], [25]]. Additionally, prior research has reported increasing hospital closures in states that did not adopt Medicaid expansion, resulting in reduced access to emergency care [26]. Previous studies have demonstrated that Medicaid expanded states had lower cardiovascular mortality rates and fewer uninsured cardiovascular disease-related hospitalizations compared to non-expanded states, especially among the uninsured [27,28]. This can be attributed to individuals in Medicaid expanded states having better access to health care across the cardiovascular disease care continuum [27].

Several limitations must be noted. First, this study cannot determine causality or the direction of association due to the study design. Second, the measure used to assess SHCB does not consider socioenvironmental contextual factors. Third, we were unable to account for individual-level CVD risk factors (e.g., physical activity, cigarette smoking). Finally, this ecological study design does not allow for casual inferences at the individual level.

## 5 Conclusion

The results of this study suggest that greater SHCB is associated with increased premature mortality due to CVD and its subtypes. Premature CVD deaths were higher in non-Medicaid expanded states compared with Medicaid expanded states among counties in the highest quintiles of SHCB. Our findings highlight the need for place-based interventions that will alleviate financial strain and expand healthcare access to patients across the cardiovascular disease care continuum.

## Funding/Support

This work was supported by the National Institutes of Health Intramural Research Program of the National Cancer Institute.

## CRediT authorship contribution statement

**Nishad S. Kosaraju:** Writing – review & editing, Writing – original draft, Methodology, Investigation, Formal analysis, Conceptualization. **Tanisha Choudhury:** Writing – review & editing, Writing – original draft, Investigation, Conceptualization. **Lee Stoner:** Writing – review & editing, Validation, Methodology, Investigation. **Aleah L. Thomas:** Writing – review & editing, Investigation, Conceptualization. **Kaitlin E. White:** Writing – original draft, Methodology, Conceptualization. **Marcus R. Andrews:** Writing – original draft, Methodology, Investigation. **Briana I. Lawrence:** Writing – original draft, Investigation, Conceptualization. **Cameron K Ormiston:** Writing – review & editing, Investigation, Conceptualization. **Lee Mason:** Writing – review & editing, Methodology, Investigation, Formal analysis, Data curation, Conceptualization. **Meredith S. Shiels:** Writing – review & editing, Resources, Project administration, Methodology, Investigation, Funding acquisition, Formal analysis, Data curation, Conceptualization. **Aldenise P. Ewing:** Writing – review & editing, Methodology, Investigation. **Yingxi Chen:** Writing – review & editing, Methodology, Formal analysis. **Jennifer K. McGee-Avila:** Writing – review & editing, Methodology, Investigation, Conceptualization. **Wayne R. Lawrence:** Writing – review & editing, Writing – original draft, Visualization, Validation, Supervision, Project administration, Methodology, Investigation, Formal analysis, Data curation, Conceptualization.

## Declaration of competing interest

The authors declare that they have no known competing financial interests or personal relationships that could have appeared to influence the work reported in this paper.

## Appendix Supplementary materials

Image, application 1
