# Supplementary material for: Severe housing cost burden and premature cardiovascular mortality
Source: Am J Prev Cardiol. 2025 Jun 2;23:101021. doi: 10.1016/j.ajpc.2025.101021 (PMC12205488; doi:10.1016/j.ajpc.2025.101021)

**Supplemental Materials**

| **Supplemental Table 1.** Cardiovascular disease mortality by subtype based on ICD-10 codes | |
| --- | --- |
| All Cardiovascular Mortality | I00-I78 |
| Ischemic Heart Disease | I20-I25 |
| Heart Failure | I50 |
| Hypertension | I10-I13, I15 |
| Stroke | I60-I69 |
| *Abbreviations:* ICD-10, International Statistical Classification of Diseases and Related Health Problems, Tenth Revision | |

| **Supplemental Table 2.** State Medicaid Expansion Status as of December 31, 2019. | |
| --- | --- |
| Expansion on or before January 1, 2014 | KY, NV, CO, OR, NM, WV, AR, RI, AZ, MD, MA, ND, OH, IA, IL, VT, HI, NY, DE, WA, CA, NJ, MN, DC, CT |
| Late Medicaid Expansion Status (Between January 2, 2014 and December 31, 2019) | NH, IN, MI, PA, AK, MT, LA, VA, ME |
| Non-Medicaid Expansion State | TN, NC, ID, GA, FL, MO, AL, MS, KS, TX, WI, UT, SC, SD, OK, NE, WY |
| Note: States that implemented Medicaid Expansion after December 31, 2019 were defined as non-Medicaid expansion state. | |

| **Supplemental Figure 1.** Age-adjusted premature cardiovascular mortality rates per 100,000, by severe housing cost burden quintiles across US counties overall and by sex, 2016–2020.    Note: As quintiles increase the larger the percentage of county households with severe housing cost burden. |
| --- |


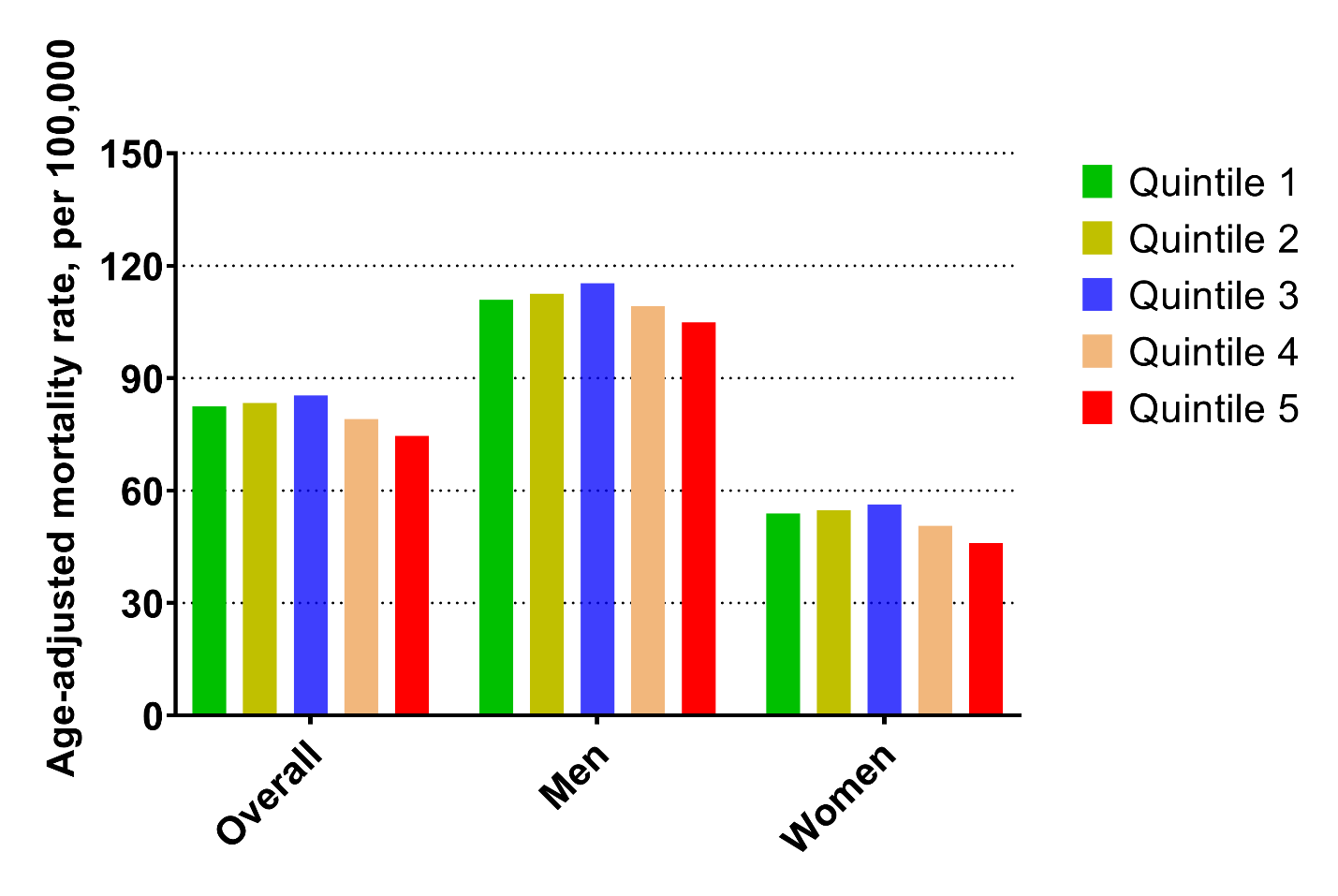


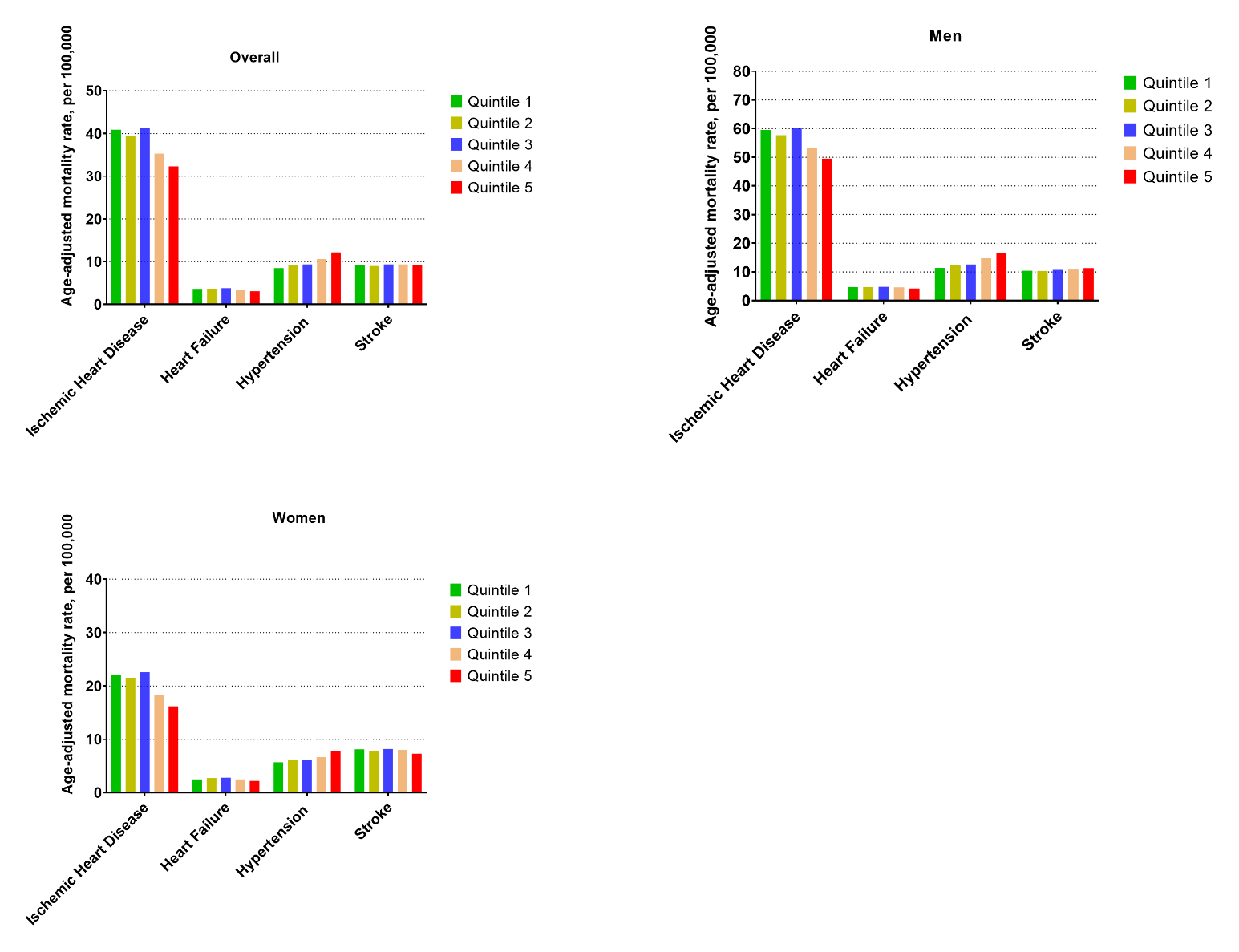


| **Supplemental Figure 2.** Age-adjusted premature cardiovascular mortality rates per 100,000, by severe housing cost burden quintiles across US counties overall and by sex, 2016–2020.    Note: As quintiles increase the larger the percentage of county households with severe housing cost burden. |
| --- |


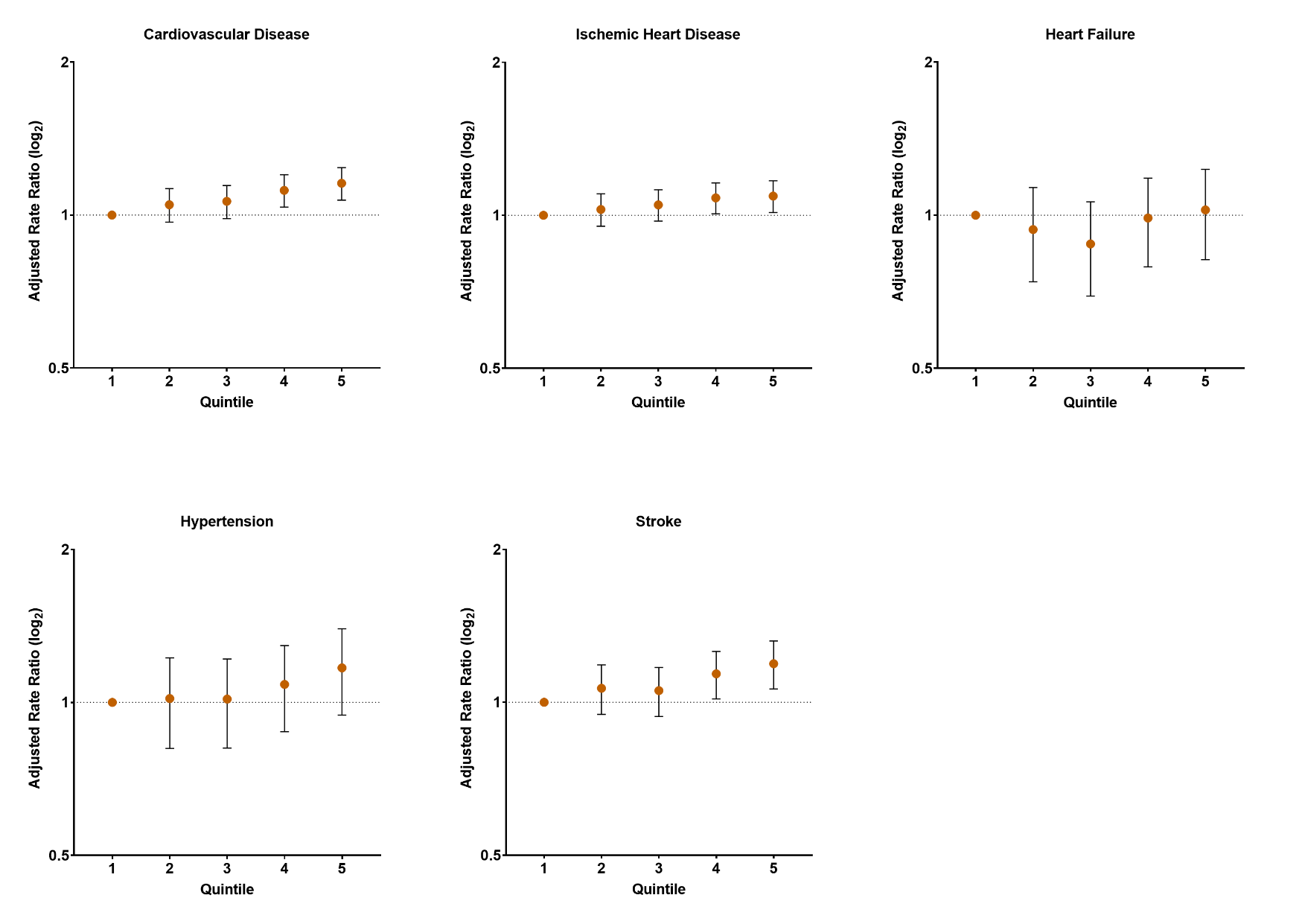


| **Supplemental Figure 3.** Adjusted Premature Cardiovascular Mortality Rate Ratios by Quintile of Severe Housing Cost Burden among men in U.S. counties, 2016-2020  Adjusted for metropolitan status, proportion 150% below poverty level, proportion of the population with non-Hispanic White alone race, proportion of uninsured adults, and education index (percent with less than high school graduate, high school only and more than high school among ages ≥25 years).  Note: The first quintile was used as the reference group  As quintiles increase the larger the percentage of county households with severe housing cost burden. |
| --- |

| **Supplemental Figure 4.** Adjusted Premature Cardiovascular Mortality Rate Ratios by Quintile of Severe Housing Cost Burden among women in U.S. counties, 2016-2020  Adjusted for metropolitan status, proportion 150% below poverty level, proportion of the population with non-Hispanic White alone race, proportion of uninsured adults, and education index (percent with less than high school graduate, high school only and more than high school among ages ≥25 years).  Note: The first quintile was used as the reference group  As quintiles increase the larger the percentage of county households with severe housing cost burden. |
| --- |


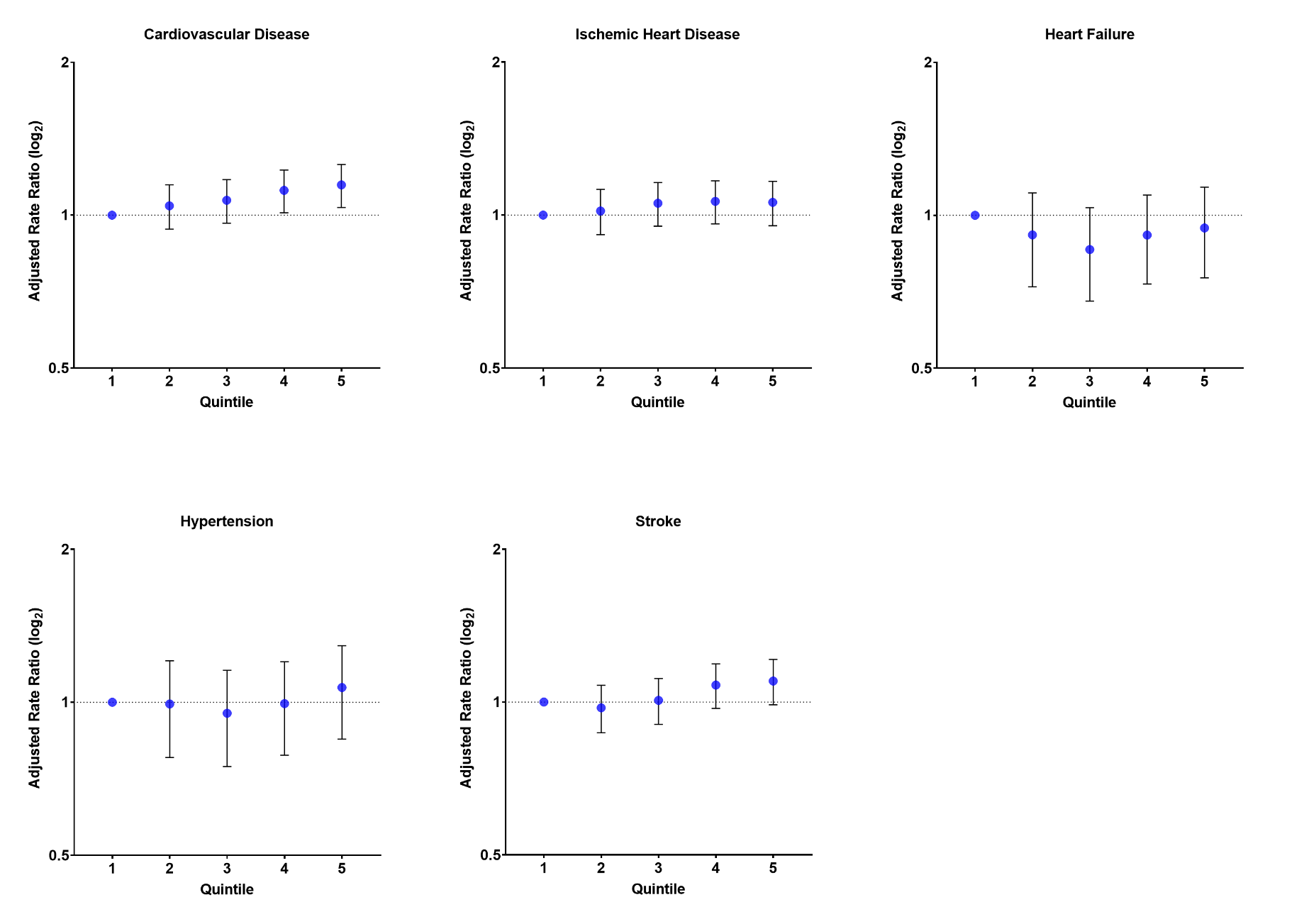

Supplement: Supplementary file 1 [file mmc1.docx]
